# The Transactions of the Provincial Medical and Surgical Association

**Published:** 1838-10

**Authors:** 


### Art. VII.

*The Transactions of the Provincial Medical and Surgical Association.* Vol. VI.—*London and Worcester,* 1838. 8vo. pp. 621.

We congratulate the members of the Provincial Association on the appearance and upon the quality of their sixth volume. We found little in its predecessor which was of interest; and, if we were consequently disposed to anticipate but little from the present volume, such anticipations have certainly been disappointed.

A Report of the proceedings of the meeting which was held at Cheltenham, July 19th, 1837, is followed by the annual Retrospective Address, delivered by Dr. Bardsley, of Manchester. A Review, one object of which is to supply the most recent intelligence of all the novelties in medicine and the collateral sciences, partakes so much of the character of a retrospective address, that it would be superfluous for us to give any detailed notice of Dr. Bardsley's very excellent performance; in which he has considered the various additions to our knowledge which have been made during somewhat more than the previous year,—has referred to the medical literature, both domestic and foreign, of that period,—has paid a merited tribute of respect to several members of our profession separated from us by death,—and has added some judicious remarks upon medical education, appropriate to our present circumstances. There are one or two facts, resulting from Dr. Bardsley's own practice, to which we may allude. In speaking of a recommendation of Dr. Prichard's, of a method of counter-irritation in certain forms of cerebral disease,—the method consisting in making an incision through the scalp down to the pericranium, in the direction of the sagittal suture, from the summit of the forehead to the occiput, the incision thus made being kept open by one, two, or three rows of peas,—Dr. Bardsley says, " I am happy in being able to offer my own testimony in favour of the practical utility of Dr. Prichard's suggestion; having myself, in two instances of severe cerebral affection, indicated by mental imbecility, stupor, and imperfect vision, adopted the plan, after the failure of the ordinary modes of treatment, and with decided advantage." Dr. Bardsley also describes, as having fallen under his own notice, "a monstrosity, apparently the effect of an abortive effort of nature to effect the formation of twins. This *lusus naturæ*, having only one head, is possessed of four arms and four legs; the sex is masculine, and the organs of generation are double, with several other analogous peculiarities," which are represented in a drawing. The child was born in the latter end of May, 1837, and was alive in July of the present year.

The second article consists of " *Introductory Observations to a proposed Plan for the Reports of Infirmaries and Dispensaries.*" A committee has been appointed by the Association for a consideration of this very important subject, of which Dr. Cowan, the author of the present observations, is the secretary. The object of Dr. Cowan is to recommend some uniform method of reporting cases; such as shall facilitate their arrangement when collected. The important statistical facts which may be obtained in this country by some systematic arrangement of the materials afforded in its hospitals may be inferred, when it is considered that " 90,000 cases are annually admitted into the British hospitals, 6,300 of which terminate fatally; and that the dispensary and out-patients amount to at least four times that number." The great utility of adopting some uniform mode of reporting these and other cases, is well shown by the difficulties attending on the arrangement of cases which have not been collected in a systematic manner. Thus, says Dr. Cowan, " M. Louis, when inspecting his materials for his work on the 'Affection Typhoïde,' was compelled to devote three whole months to rewriting his own reports, for the purpose of arranging them on some uniform plan, by which their comparison became possible. Now it is evident that his labour might have been avoided, or materially lessened, had a correct method of reporting been originally laid down." And it is likewise said that " M. Chaponiere, in his inaugural thesis at Paris, 1832, on 'Facial Neuralgia,' spent several months in collecting all the observations he could find in the extensive library of the school, and from other sources; but, of 653 recorded cases, he was obliged to reject 400, in consequence of the irregular and incomplete manner in which they were reported."

Dr. Cowan has suggested a tabular arrangement, which is intended to direct the attention of members more distinctly to the subject. It is not put forward as perfect; as doubtless some members may be able to suggest particulars in which it may be improved: and as it is hoped that whatever arrangement is decided on will be pretty generally adopted, a scheme as perfect as possible is to be desired.

The next paper is "*On the Medical Topography of Exeter and its Neighbourhood; being a Sketch of the Geology, Climate, Natural Productions, and Statistics of that District.* By Thomas Shapter, M.D." We cannot speak too highly of this very elaborate performance: it seems to leave no attainable point unreached. It is in vain to attempt a detailed analysis of Dr. Shapter's memoir, but we cannot deny ourselves the gratification of a brief outline of it.

The portion of Devonshire to which the paper relates is Exeter and its immediate neighbourhood. The river Exe flows through the city, and, between this river and its opening into the sea, various towns, it is well known, are situated. The mean height of the land boundaries to the south-west of the district is 1,782 feet; to the north, 740 feet; and there are also high lands to the east. The general physiognomy is such as is seen in countries with undulating high grounds and luxuriant and highly cultivated valleys. Exeter occupies the flat summit and declivities of a hill which rises from the eastern bank of the river. The summit of the hill is 149 feet above the level of the sea. The hilly character of the situation favours cleanliness and ventilation. The western part of the city is occupied by the great mass of the poor: much of this portion is situated on the steep declivity, running westerly downwards, and thus affording sufficient drainage and ventilation; the remainder of this quarter is a hollow flat. The south-eastern quarter is occupied by the resident gentry, and is open, pleasant, and healthful. The northern and southern quarters are occupied by persons in moderate circumstances. The general character of the surface and soils in the district varies considerably; ranging from the granitic, through the cold slaty soils, to the fine, rich loam lands on the sandstone. The city, by means of its river, wells, conduits, springs, &c., is well supplied with water. Dr. Shapter has mentioned the leading characteristics of all the waters in the district: they consist of common hard spring-water; some of them, however, containing a sufficient quantity of mineral impregnation to entitle them to be called mineral springs; and of acidulous chalybeate, which also, in some instances, might, from their peculiar impregnations, be termed mineral waters. Notwithstanding the general character which Exeter and its neighbourhood have obtained for mildness and equableness of temperature, there is very little certain information recorded respecting these points. The averages from which the following estimates are made "are, in almost every instance, deduced from observations made during a period of ten years,—i. e. from 1825 to 1834 inclusive,—and are contrasted with those deduced from registers kept in the metropolis;" a system of comparison which has been already adopted in the Society's Transactions.

The *annual mean temperature* of Exeter is 51°29; of London, 50°39: and in this calculation Dr. Shapter is of opinion that the temperature of London is too high, since the London average is derived from registers kept in the heart of the metropolis, where the climate is influenced by the circumstances of a great city. Some tables given by Dr. Shapter tend somewhat to support a statement made by Luke Howard, that there is an ascending and descending series of temperature, rising and falling by alternate years, rendering it probable that we have in this island a cycle of temperature, in which the climate becomes warmer and colder by turns, in such a way as to exhibit both extremes in the space of seventeen years.

The *mean temperature of the seasons* is as follows:

| | Winter. | Spring. | Summer. | Autumn. |
|---|---|---|---|---|
| Exeter........ | 41.80 | 49.51 | 62.08 | 51.94 |
| London ...... | 39.1 | 48.7 | 62.3 | 51.3 |

The *absolute range* is

| | Greatest. | Lowest. | Mean. |
|---|---|---|---|
| Exeter......... | 94 | 16 | 70 |
| London....... | 96 | 5 | 91 |

From the recorded tables it may be seen that the climate of Exeter and its neighbourhood, though not so equable as that of Penzance, is justly worthy of its character as to mildness, for which it is so justly esteemed. As contrasted with London, the *equability* of the climate is very marked; since, although the mean temperature of Exeter is greater than that of London, yet the summer of the latter city has a higher temperature, whilst its winter is considerably lower. By comparing his district with other places within the same zone of temperature, and by drawing an isotherial line, and regarding how much the mean temperature of the places it cuts is in excess of that of Exeter, Dr. Shapter has further shown the comparative equability of the temperature of his district.

The quantity of *rain* which falls on the Devonshire coast is almost proverbial; but Exeter itself seems to be unjustly suspected of being very rainy. The mean annual fall, in inches, is as follows:—Exeter, 29.12; St. Thomas, 31.90, (this is a parish in the vicinity of Exeter;) London, 25.00. But the average number of wet days (by which is meant a day on which a fall takes place, however slight it may be,) is in favour of Exeter: e. g. St. Thomas', 162.4; London, 178. The least rainy season is the spring; the autumn is the most so in regard to quantity; but in the winter is the greatest number of rainy days.

Continued *frost* is very rare; although Dr. Shapter states that, about once in five years, "it continues sufficiently long to freeze the river to a state thick enough to sustain large masses of people." In ten years, there were sixty-nine days during which *snow* and *sleet* fell; and *hail* is scarcely more frequent than snow. As compared with London, the relative proportion of *winds* for ten years is as follows:

| | N.W., W. | S.W., S. | S.E., E. | N.E., E. |
|---|---|---|---|---|
| Exeter, 1825-34 .. | 131.3 | 86.2 | 71.1 | 75-6 |
| London, 1807-16 .. | 100.4 | 104.4 | 53.9 | 74.4. |

The barometer is highest during the N.E. and N. winds, and at the lowest during the S.W. and S. winds. The thermometer is at its maximum average during the S. and S.E. winds, and at its minimum during the N.E.: but this last wind, which has the least elevated temperature during the winter months, becomes the warmest in June. During the winter season, the s.w. wind is frequently accompanied by a warm, thick mist, which has a peculiar relaxing softness in its feel, and is not unaptly styled the true Devonshire weather. The list of exotics capable of cultivation in the open air is further confirmatory of the mildness of the Exeter winters.

Dr. Shapter next gives an account of the supply of the markets; of the natural productions of the district; of its civil and economical history, &c. The agricultural population forms only 1-94th part of the whole, and rather more than 1-6th are engaged in trade and handicraft. There appears to be nothing peculiar in the *occupation* of the inhabitants worth dwelling on. Dr. Shapter thus describes the cider which the Devonshire labourer calls good: "It is full in body, rough in taste, and hard, and has no flavour of the apple remaining in it. The sweetness of the cider prepared for exportation is preserved by a process to which it is submitted during its manufacture, termed 'matching.'" Since lead has been almost entirely discontinued from among the utensils used in the cider manufacture, Dr. Shapter states that the *colica pictonum* is no more frequent in Devonshire than in other countries.

The second part of Dr. Shapter's paper is *Statistical and Medical;* but it is from "the concluding portion of this paper, embracing the general law of mortality, together with the medical history and medical statistics of the city," and which "will follow in a subsequent volume," that we shall expect to derive facts of greater interest to our readers. The statistics here introduced are such as are only illustrative of the effect of climate. Tables which are 'the result of very careful and elaborate examinations of the different parish registers" are subjoined, to exhibit the data whence the average mortality is deduced. As this subject will be further treated of, with the Law of Mortality, we pass it by at present, as well as another series of tables, which "place in apposition the climate, with the mortality of those ten years which throughout this paper have engaged our attention."

We can safely recommend to his fellow associates Dr. Shapter's paper, as a model to be followed in prosecuting the great object of the Association, the formation of a complete Medical Topography of England.

The fourth article is by Mr. Nash, "*On the Medical Topography and Statistics of Cheltenham.*" This paper is not, as the former, the result of long and careful observation; yet it is very creditable to the author. The medical department of its statistics is, however, of very little value. There is more which is interesting in the geological and botanical history of the place, as also in the account of its mineral waters; but our limits prevent us from entering on these subjects. We notice one or two particulars of the climate, for the sake of comparison with what we have recorded respecting Exeter. The annual mean temperature of Cheltenham is 50°.26, calculated on an average of seven years. The mean temperature of the seasons is as follows:—Spring, 49°.65; summer, 60°.80; autumn, 50°.28; winter, 40°.92. The average number of days on which rain has fallen is 110; and the annual fall of rain (during four years) 33 inches. There seems little difference in the character of the winds from that of the more southern and western parts of the island. There are three maps associated with Mr. Nash's paper: a geological map of the country around Cheltenham; a section of the vale of Gloucester, from the Cotswold hills to the river Severn; and a map of the number of feet above the river Severn of Gloucester and the neighbouring country.

The Third Part of the volume, consisting of "*Essays and Cases*," commences by a most accurate and interesting "*Cursory Examination of the Works of Galen, so far as they relate to Anatomy and Physiology*," by Dr. Kidd, of Oxford. It is greatly to be wished that Dr. Kidd would take the trouble of making similar analyses of the works of other ancient medical authors: no one is so well qualified for the task. To many practitioners the works themselves are inaccessible; to many more neither time permits nor inclination leads to their perusal; but essays such as the present are at once accessible and acceptable to all. We would gladly transcribe the whole, but must limit our notice to a few extracts.

The system followed by Dr. Kidd is, "after a few introductory remarks, to examine, first, Galen's account of the organs and processes of nutrition, secretion, and reproduction; then of the instruments of voluntary motion; and, lastly, his account of the nervous system." Most of the descriptive terms, both in physiology and pathology, as well as in anatomy, which are now in use, were employed by Galen in the same sense as they are employed by modern authors. He appears to have been possessed of great anatomical dexterity. He regarded the stomach, intestines, liver, and veins as the organs of nutrition. The veins were said to convey the chyle from the stomach and intestines to the liver, by the vena portæ; the formation of which, and its several branches, are very well described. The venæ cavæ hepaticæ were regarded as the commencement or root of the venous system of the body at large. Galen seems to have known the dependence of the condition of ulcers of various parts upon the process of digestion: he observes that, if food be unwholesome, the resulting chyle will not be good, and that from unhealthy chylification, phagedenic and other ulcers will arise. He was not aware of the true nature of the lacteals; but he spoke of the convolutions of the intestines being numerous, in order that the surface for absorption might be extensive. He considered that the blood was perfected in the liver. Whilst he admitted, according to the prevalent theory, that the arteries contained air, he asserted at the same time that they naturally contain blood also; for he says that, if an artery be wounded, blood immediately flows from it, without any previous escape of air; which is a proof that blood was already in the artery: for, had it made its way into the artery from any other source, the air must have first escaped. With reference to the decision of the general question, whether blood is contained in the arteries, he relates the following anecdote:

"There are some teachers," he says, "who are in the habit of advancing opinions which they are not prepared, and therefore not inclined, to put to the test. Such was the case with a certain teacher of anatomy, who, having declared that the aorta contains no blood, and having been earnestly desired by some of the ardent pupils of Galen to exhibit the requisite demonstration, they themselves offering animals for the experiment, declined, after various subterfuges, to satisfy them without a suitable remuneration: on which the pupils immediately raised a subscription among themselves for the purpose, to the amount of a thousand *drachmæ*, (equivalent, probably, to about twenty-five or thirty pounds of our money.) The professor, being thus compelled to commence the experiment, failed in the attempt to cut down upon the aorta, to the no small amusement of the pupils; who, thereupon, taking up the experiment themselves, made an opening into the thorax, in the way in which they had been instructed by Galen; passed one ligature around the aorta at the part where it attaches itself to the spine, and another at its origin; and then, by opening the intervening portion of the artery, showed that blood was contained in it."

Galen knew that, notwithstanding the force with which blood is propelled from a wounded artery, gentle pressure of the finger is sufficient to stop its further escape; and that, during the effect of this pressure, the blood soon coagulates, and forms a natural plug within the artery. He regarded the whole arterial system as consisting of those pulmonary vessels which end in the left auricle, together with the aorta and all its ramifications; and he compares these pulmonary vessels, in their supposed office of attracting air from the lungs, to the roots of a tree, which attract nourishment from the surrounding soil; and the aorta and its ramifications, which are supposed to receive the air forwarded by the pulmonary vessels, to the trunk and branches of a tree receiving its nutriment from the roots. The veins are considered by Galen as the efficient nutrients of the body at large. He had a considerable acquaintance with the uses of the valves of the heart: he knew that the heart was the source of pulsation; for he says that, if a ligature be made on an artery, pulsation continues in that part of the artery which is intermediate to the *ligature* and the *heart;* but ceases in that part of the artery which is intermediate to the *ligature* and the *extremities.* But he cannot explain why nature, who does nothing without design, should have made different vessels, arteries, and veins, to contain the same fluid; and he has no knowledge of the circulation, although acquainted with the anastomosis of these two orders of vessels. He describes two kinds of respiration; one by the mouths of the arteries of the lungs, and one by the mouths of the arteries of the skin: in each case the surrounding air is drawn into the vessels during their diastole, for the purpose of cooling the blood; and, during their systole, the fuliginous particles derived from the blood and other fluids are forced out. He supposes that the cuticular respiration is sufficient for animals during hybernation. Galen regards the diaphragm as the chief agent in ordinary respiration, as distinguished from the forced respiration which precedes any violent muscular effort; and he regarded the heat of the blood as in part being produced by respiration. He speaks of a quality in the air which is inimical to life. He describes various effects produced by dividing nerves, and shows the effect of dividing the recurrent branch of his sixth pair of cerebral nerves (the pneumo-gastric of modern anatomy;) explaining why the division of this branch, though it be made on both sides, does not entirely destroy the voice: he also explains how it happens that, if the spinal marrow be divided *beneath* the lower termination of the neck, the diaphragm will still continue to act; because the origin of the phrenic nerve is *above* the lower termination of the neck, In answer to some contemporaneous physiologist, who asserted that urine is only the excrementitious portion of that blood which nourishes the kidneys, Galen gives a good specimen of just physiological reasoning; for, from the very large size of the renal vessels, compared with the size of the kidneys themselves, he argues that those vessels were not intended solely for the nourishment of the kidneys, being capable of affording nourishment to organs of a much larger size; and he therefore concludes that, from the blood contained in them, not only are the kidneys nourished, but the urine also is secreted. His opinion on the nature and source of the catamenial discharge accords generally with that of modern physiologists. The discharge proceeds, he says, from the inner surface of the uterus, through the same orifice in its neck by which the embryo passes. In its character he considers it as somewhat different from the blood; observing that, at the period of the catamenia, the inner surface of the uterus is bathed by a moisture of a sanguineous nature, which has been secreted from the blood. Galen remarks that sensation and voluntary motion essentially distinguish animals from plants; and that, on this ground, nerves constitute muscle an *animal* organ, which, with reference solely to its arteries and veins, &c., is a merely *physical* organ. He supposes, however, that nerves do not possess an inherent and independent power, but that whatever power they have is derived from the brain; from which, though they have no perceptible cavity within them, they are capable of conveying sensation and power of motion to the most distant parts of the body. He distinguishes between the powers of voluntary and involuntary motion; and observes, that those organs of sense which are obedient to voluntary motion, as the eyes and the tongue, require, consequently, two sets of nerves; and, with respect to the tongue, he points out those nerves which belong to it as an organ of taste, and those which render it capable of voluntary motion. His opinion was also that the functions of the nerves arising from the spinal marrow were dependent on the brain. In practice he saw the importance of a knowledge of the parts of the spinal marrow to which ramifications of nerves belonged. Thus, when called to a patient who had for some time lost the sense of feeling in some of his fingers, and had derived no benefit from local applications to the fingers themselves, Galen enquired whether he had ever met with any injury of the spine; and, having been informed that he had fallen on his back some time previously with considerable violence, Galen applied remedies to the part on which he had fallen, and thus restored sensibility to his fingers. He relates experiments which he made in order to prove that, by the division of the spinal marrow in various parts, both the power of motion and sensation are destroyed in those muscles which are connected with the divided parts.

But we must, however unwillingly, terminate our abstract of Dr. Kidd's very interesting paper. As an indulgence of literary curiosity, if no other benefit is to be derived from an acquaintance with them, we should much like to see a similar analysis of Galen's works, "On the Differences and the Causes of Diseases," and "On the Method of Cure," together with other of his productions more immediately relating to medicine.

The next article, "*On the Treatment of Hypertrophy of the Heart, and chronic or subacute Inflammation of the Pericardium, especially in reference to the beneficial use of small Doses of Mercury in those Affections,*" by Mr. Salter, of Poole, is one of a purely practical character. It is a most valuable essay, characteristic throughout of a thoughtful and rational mode of pursuing practice, and of the benefits likely to result from the opportunities of communication afforded by the Provincial Association. The paper is so valuable that we scarcely like to abridge it; and it is, therefore, a matter of great satisfaction that, through the medium of the Association in whose published Transactions it appears, it will fall under the notice of a large number of the profession.

The title of Mr. Salter's paper might have been more comprehensive, since the treatment recommended is said to be "applicable to all varieties of chronic diseases of the heart, with the exception of simple dilatation of its cavities, called by Corvisart, 'passive aneurism of the heart.'"

The greatest number of cases treated by Mr. Salter have been instances of hopeless organic disease, where palliation only could reasonably be looked for. Respecting these, however, he says, "I trust that I shall be able to show that we possess the means of staying the progress of morbid action, to an extent that has not commonly been thought possible; and that those persons who have the misfortune to labour under incurable disease of the heart may, notwithstanding, in a considerable degree, be rendered capable of enjoying the pleasures of life."

In considering the pathology of cardiac diseases, it is necessary to bear in mind the congestive states of parts influenced by the impeded circulation, such, for instance, as the lungs and liver; and these require attention in treatment: and it is remarked "that the means which are found effectual in removing the primary complaint are often the best suited for such as are superinduced by it; and this applies more especially to the prudent exhibition of mercurials." The influence of mercury in checking acute inflammations of the heart is well known; but Mr. Salter remarks that "it has remained to be shown that a modification of the mercurial treatment is a powerful means of checking the progress of those slow organic changes, which, if they do not destroy life so rapidly, are nevertheless as certainly fatal as the more active diseases, if allowed to go on uncontrolled by efficient treatment." We believe, with Mr. Salter, that, notwithstanding it has been the practice with some individuals to administer mercury in the forms of diseased heart to which he alludes, it remained to be shown how very extensively applicable it was in this class of affections. Mr. Colles, in his work on the Use of Mercury, alludes to the practice in the following terms, which we are happy to quote as confirmatory of the practice recommended by Mr. Salter. At the same time we are aware that, when Mr. Salter's communication was written, Mr. Colles' book was not published. "In cases of acute inflammation of a joint, or of the dense membranes of the eye, we find" says Mr. Colles, "that the progress of the disease is arrested the moment that the salivary system becomes affected; and even in cases of other diseases, which cannot be considered as purely inflammatory or acute, the same remark will be found to hold good: thus, in cases of orthopnœa, depending on organic disease of the heart, with effusion into some of the thoracic cavities, and in which we commonly prescribe mercury in combination with squill and digitalis, the patient is not at first sensible of any improvement, but almost invariably, as soon as the gums become affected, he experiences relief; and, perhaps the very next morning after this occurrence, he tells us, with joy and gratitude, that he is considerably better, that he has passed a night of refreshing sleep, and that he has been able to do what he could not have done for weeks previously,—namely, rest in the recumbent posture, without any of that alarming and distressing sense of suffocation under which he had previously laboured, and which always supervened the moment he sunk into that position." (pp. 31, 32.) And, again, at page 48, it is remarked that, in "organic affection of the heart, attended with orthopnœa and effusion into the chest, the ptyalism, established on the sixth or seventh day, may be kept up without injury for ten or fifteen days longer, if the obstinacy of the symptoms should require it," &c.

In addition to the acute and chronic forms of *pericarditis* generally described, Mr. Salter infers, from his own observations, that there is a milder or subacute form, consisting in a condition of hyperæmia. This, he observes, is mostly unattended with effusion or sensible thickening of the membranes; is commonly, though not always, a secondary affection accompanying chronic diseases of the heart. The condition above mentioned is regarded as receiving its best illustration from phenomena observed in an organ possessing an analogous structure, the eye. In this, especially in rheumatic subjects, a condition of hyperæmia often exists, which cannot be said to amount to inflammation: and this state may exist for a long time, and, when removed by proper management, will recur from the patient taking cold, or from a disordered condition of the digestive organs. Even this minor degree of congestion existing in the heart will, as is plainly demonstrated in the eye, if uncontrolled, lead to the most serious consequences, and may at any time quickly pass into a morbid action of the vessels of a more active kind. The inflamed state of the pericardium here referred to gives rise to the tenderness between the ribs in the cardiac region and beneath the ensiform cartilage, observed in chronic cardiac affections.

Although we are disposed to agree with Mr. Salter in thinking that there may exist some condition similar to that above mentioned, it is one which is without sufficient evidence. Mr. Salter speaks of this condition as being " mostly unattended with effusion or sensible thickening of the membrane." But these are negative signs; and it is not pretended that any congested state of the pericardium has actually been witnessed. Indeed, we are disposed to think that, in some of the cases in which the mercurial treatment has been so beneficial, it may have been by benefiting a condition of endocarditis, as well as of pericarditis or congestion of the pericardium: and of this endocarditis or (if an inflammatory nature does not belong to the condition to which we allude,) anormal state of the capillaries of some parts of the membrane lining the heart, we have probably evidence in the various changes existing in the valves. But this is a question more of curiosity than of any practical importance.

The treatment employed by Mr. Salter is as follows:—If there appears to be an absolute or relative plethora, some venesection precedes the use of mercury. The blue pill is then employed; the object of its use "being to make the gums slightly tender, as soon as it can be done, and with the smallest possible quantity of the mineral, and to keep up very moderate mercurial action for some little time after the symptoms have been relieved." Of the effect of this treatment Mr. Salter thus speaks:

" I have seen patients affected with orthopnœa, unable, but for a short period, to bear the recumbent posture, and scarcely able to walk across their chambers, or to stoop to tie their shoes, labouring also under anasarca of the feet and legs, lose in a week all the general symptoms of their disease, walking about apparently in their former health; and this benefit has been brought about without any evident operation of the medicine, besides its influence upon the mouth." (p. 345.)

Where there is much congestion of the bronchial mucous membrane, preventing the due decarbonization of the blood, and when the circulation of this blood through the brain adds to the nervous irritation, and prevents rest, bleeding is recommended; but in such a posture of the patient that, on the approach of syncope, he may immediately lie down. This depletion alleviates the state of congestion and oppression of the chest, and gives time for the establishment of mercurial action, and it also contributes to remove a disease (bronchitis), which of itself is often highly dangerous. We must quote the following remarks on the connexion between cardiac disease and inflammation or congestion of the mucous membrane of the bronchi in the author's own words, because of their great practical importance, more than on account of any novelty which they contain. (p. 346.)

"I am convinced, from attentive observation, that death is frequently hastened, if not produced, either by failing to notice, or not actively treating, the morbid condition of the bronchial mucous membrane existing in cardiac diseases. Cold, we know, acts as a stimulus to the pulmonary mucous membrane; and the disordered state of this tissue in heart diseases increases its susceptibility to be impressed by atmospheric vicissitudes. Persons, not aware of this fact, by exposing themselves in the winter season, often put their lives in imminent danger. For the same reason, epidemic influenza becomes highly hazardous to individuals suffering from disease of the heart. So decidedly am I impressed with the correctness of these views, that I consider it much more important to this description of patients to be placed, in the winter, under the influence of a regulated warm temperature, than to the truly consumptive."

In the mild cases of bronchitis, complicating organic disease of the heart, Mr. Salter considers that the mercury alone may be trusted to; but he does not wish to be regarded as recommending mercurialization for the cure of inflammation of mucous membranes generally. If the dropsy attending these cardiac diseases do not materially interfere with any vital functions, it may safely be left to the influence of the mercury: this drug, in these disorders, often proving the most efficient diuretic. In worse cases, Mr. Salter has found elaterium preferable to digitalis. Elaterium is most safely given when there is firmness of the anasarcous swelling. If the limbs be soft, doughy, and transparent, something more mild should be tried; and, when the elaterium has carried off the dropsical effusion, the use of mercury should be immediately commenced, and cautiously continued until the gums are slightly sore; and this action should be kept up until the breathing is decidedly relieved, and for some short time afterwards, and should then be had recourse to on a recurrence of the difficulty of breathing. In this manner, by the use of elaterium, mercury, and (if in the winter) confinement to the house in rooms whose temperature should never be below 50° F., the complaint may be kept at bay, and the fatal event warded off for a very long period. The relation of six cases, chosen from among many others, illustrative of the benefits of this treatment, terminates Mr. Salter's communication. They are all well worthy of perusal, and strongly confirm the recommendation of ptyalism as a palliative means in organic diseases of the heart.

The next paper is by Dr. England, of Wisbeach, and contains an account of "*two Cases of Gangrene of the Lungs,*" which presented nothing very novel. This is followed by a very interesting relation, by Dr. O'Bryen, of Bristol, of "*a Case of partial Ectopia Cordis and Umbilical Hernia,*" which, however curious, and indeed, we believe, unique, must give place to matter of a more practical kind. The case is well worthy the attention of both physiologists and pathologists.

This part of the volume terminates with the relation of an interesting and successful case of "*Extirpation of the Eye, on account of a Tumour within the Optic Sheath,*" by Mr. Middlemore.

The next two articles are "*Reports of Out-Patients treated in the Birmingham Town-Infirmary,*" by Mr. T. Ryland and Mr. S. Berry. These appear to have been drawn up with great care. From the former report we may abridge a case of *Strangulated Hernia,* as encouraging hope under what appears to be the worst circumstances. The case was that of a stout widow, æt. 86, who had been subject to femoral hernia, on the right side, for twelve months. 13*th March:* For the first time it gave her pain, and suddenly increased in size. Vomiting occurred, and the bowels became painful and constipated. 17*th:* Mr. Ryland first saw the case: the hernial tumour was as large as an orange, tense, rather sore, and quite irreducible. Bowels were not distended, and had been slightly opened on the 14th; tenderness in the right iliac region. A castor-oil enema brought away a large quantity of fæces; everything swallowed produced vomiting.

The female would not submit to an operation, which, although offering comparatively small hopes of a successful issue, it was considered right to recommend. She continued to vomit everything swallowed, together with a large quantity of fæcal matter. In this state she continued for many days, getting gradually weaker, but suffering no pain from the hernia, and only a little occasional uneasiness in the bowels. On the twenty-fourth day of the strangulation she complained of being famished to death; she therefore ate heartily of bread and cheese, and drank beer with much relish. On the following morning she passed flatus, and soon afterwards a large quantity of fæces, which, in the course of the day, amounted to a chamberpot full. She was weak, but had not vomited since the first motion; the hernial tumour was undiminished. She took gin and water in small quantities, and recovered.

Dr. Ogier Ward also gives an excellent "*Report of Cases treated at the Birmingham Dispensary.*" He suggests, as an interesting question, why, in the present report, as well as in all those which he has examined, the proportion of deaths in males is so much greater than in females; while the cases of disease in females are so much more numerous? In speaking of the cases of *Amenorrhœa,* Dr. Ward mentions that they were all attended with more or less headach, and that, in the treament of this symptom, he was guided less by a regard to the constitution of the patients than by attention to the effect of the recumbent posture in alleviating or aggravating the pain: such patients as are relieved by lying down being almost invariably benefited by antispasmodics and tonics, particularly iron; while those who sleep with the head raised, and whose eyes are swelled in the morning, require depletion, local and general. In two cases of *Anæmia,* the *bruit de diable* was observed; a symptom which

Dr. Ward has lately noticed in many cases, not only of anæmia, but of general debility, in which it was conjoined with palpitations, in women past the menstrual period. He has not met with the *bruit de diable* in a male patient; and he alludes to the fact of the British Association having confirmed his opinion as to the venous origin of the sound; though he doubts the correctness of their statement that they have observed it in the arteries also, believing that, in such cases as appear to justify this conclusion, the sound was caused in the veins accompanying the arteries. A case is related (p. 440,) in which it appears that *perforation of the intestines* occurred five weeks before producing fatal effects. The case is very interesting, although not absolutely justifying the inference that the perforation had existed for so long a period; but it is too long to extract, and should not be abridged.

There are many very interesting facts in Mr. Middlemore's "*Report of Cases, attended during the year* 1837, *at the Birmingham Eye Infirmary*." One of these is that of an ossified capsule of the crystalline lens, which was removed through a section in the lower part of the cornea. "The anterior hemisphere of the capsule was almost entirely converted into a smooth plate of bone, except near the margin of the union between the anterior and posterior hemispheres of the capsule, where it constitutes a ragged ring of bone." We subjoin the following remarks on "fistulous opening communicating with the anterior chamber." A cork struck a woman in the eye; a few days after which was found a small, nearly transparent tumour, just without the margin of the cornea, which contained a small quantity of aqueous fluid. On its removal by a minute needle opening, it soon reappeared, and the iris appeared somewhat narrower on the side of this vesicular enlargement. No astringents effected its contraction, and, on opening it again with a fine needle, it soon refilled. It afterwards gradually diminished on the application of nitrate of silver, and it has not since reappeared.

Mr. Middlemore has tried the effects of various local means, much vaunted of late, in certain affections of the eyes; and he communicates the results of his experiments as follows:

*Veratrine and Aconitine Ointment.* In only one case of amaurosis, out of eight subjected to the treatment, was the slightest benefit obtained—in the case of a soldier suffering from dimness of vision, which, however, was not so great as to prevent him from walking about the streets of Birmingham, and managing the sale of vegetables at home. The pupil of the eye was rather large and sluggish, and there was just that sort of muddy green appearance within the eye which is noticed where chronic inflammation of the septa of the hyaloid membrane has induced a slightly turbid condition of the vitreous fluid. The pupil (and Mr. Middlemore mentions this as a very common effect of these applications,) became smaller and more active; and he thinks his sight considerably improved. After discontinuing the remedy, his sight was very little better than it was before he used it: (Mr. M. does not say which of the above ointments he employed in this case.) In some cases of neuralgia of the eyeball, he has used these ointments with unequivocal advantage. They consist of four grains of aconitine or veratrine to half an ounce of lard. A quantity, the size of a *small* nut, is to be rubbed above the eyebrow, by means of a bit of sponge attached to a convenient handle, until the skin begins to smart and feel very hot: the rubbing to be practised daily.

As we know that the recommendations lately given to apply the solid nitrate of silver to the eye in some states of disease have led to a destructive mode of using it, and are likely, in the hands of rash practitioners, to lead to still further mischief, we subjoin Mr. Middlemore's mode of using the Nitrate of Silver, in certain forms of amaurotic affections: "Having a portion of nitrate of silver worked to a delicate point, I touch the cornea near its junction with the sclerotica, so slightly as merely to produce a small eschar; on the detachment of which a minute, superficial, and perfectly healthy ulcer remains, which very readily heals and becomes imperceptible; and this I do at about four points, which are comprised within the half of the cornea." *Use of Strychnia:* In two cases of amaurosis, after the failure of the other methods, a blister was placed in the front of the ear, and a small quantity of strychnia was applied to the raw surface left after its removal, with the most satisfactory results; especially satisfactory on account of the success of this after the failure of other methods of treatment.

Our limits will not permit us to notice the last article, which is a "*Statistical Account of the In-Patients of the Medical Wards in the Geneva Hospital, for the years* 1834, 1835, *and* 1836: *to which are added, Documents relative to the Influence of the Seasons on the Development of certain Diseases amongst the poorer Classes of Geneva and its Environs.* By Dr. Lombard, Physician to the Civil and Military Hospital of Geneva." It is, however, a very valuable paper, worthy of its distinguished author, and showing the benefits derived by the Association from its corresponding members.

The volume terminates with a "*Report upon the Influenza or Epidemic Catarrh of the Winter,* 1836-7," drawn up by Dr. Streeten, of Worcester, from the communications of numerous members residing in various parts of England, in reply to a set of queries issued by the Council. This is a paper of so much value, and the subject is so interesting, that we purpose devoting a separate article to its consideration.

---

